# Evaluation of a thrice weekly administration of teicoplanin in the outpatient setting: a retrospective observational multicentre study

**DOI:** 10.1093/jacamr/dlab012

**Published:** 2021-02-21

**Authors:** John Asumang, Katie L Heard, Oliver Troise, Sandra Fahmy, Nabeela Mughal, Luke S P Moore, Stephen Hughes

**Affiliations:** School of Medicine, Imperial College, London, SW7 2DD, UK; Chelsea and Westminster NHS Foundation Trust, 369 Fulham Road, London SW10 9NH, UK; Chelsea and Westminster NHS Foundation Trust, 369 Fulham Road, London SW10 9NH, UK; Chelsea and Westminster NHS Foundation Trust, 369 Fulham Road, London SW10 9NH, UK; Chelsea and Westminster NHS Foundation Trust, 369 Fulham Road, London SW10 9NH, UK; Imperial College London, South Kensington, London SW7 2AZ, UK; North West London Pathology, Imperial College Healthcare NHS Trust, Fulham Palace Road, London W6 8RF, UK; Chelsea and Westminster NHS Foundation Trust, 369 Fulham Road, London SW10 9NH, UK; Imperial College London, South Kensington, London SW7 2AZ, UK; North West London Pathology, Imperial College Healthcare NHS Trust, Fulham Palace Road, London W6 8RF, UK; Chelsea and Westminster NHS Foundation Trust, 369 Fulham Road, London SW10 9NH, UK

## Abstract

**Introduction:**

The glycopeptide teicoplanin is commonly utilized to facilitate outpatient parenteral antimicrobial therapy (OPAT). Licensed for once daily maintenance dosing, teicoplanin’s long half-life allows for less frequent dosing (e.g. thrice weekly) following successful loading. This service evaluation reviews the safety and effectiveness of a novel thrice weekly teicoplanin dosing regimen.

**Methods:**

A retrospective, observational study was conducted at Chelsea and Westminster Hospital (March 2018 to July 2020), evaluating trough serum teicoplanin concentrations for patients receiving >5 days of teicoplanin in the OPAT setting. Teicoplanin dosing and administration (once daily versus thrice weekly), clinical outcomes and therapeutic levels were analysed for all patients. The project was registered with clinical governance locally.

**Results:**

A total of 82 patients treated with teicoplanin in the OPAT service were included; 53/82 receiving thrice weekly and 29/82 receiving once daily dosing. Mean teicoplanin trough levels were similar in both groups (26.2 mg/L and 25.8 mg/L in once daily and thrice weekly groups, *P *=* *0.8895). High clinical success rates were recorded in both groups (25/29 [86.2%] versus 50/53 [94.3%]). No correlation with clinical outcomes and initial teicoplanin serum levels was identified. Normal renal function (>90 mL/min) was associated with lower teicoplanin serum concentrations (mean [±SD] 21.4 mg/L [±10.1] versus 29.7 mg/L [±14], *P *=* *0.0178) in the thrice weekly dosed group but not with the once daily dosed group (mean [±SD] 28.2 mg/L [±9.4] versus 23.7 mg/L [±9.9], *P *=* *0.2201).

**Conclusions:**

This study supports thrice weekly teicoplanin as a convenient and effective OPAT for administration in the OPAT setting. Therapeutic drug monitoring is advised to adjust for intra-patient variability.

## Introduction

Teicoplanin, a glycopeptide antibiotic, is licensed to be administered via once daily (OD) IV or intramuscular (IM) injection following appropriate loading. Patients receive an initial ‘loading’ dose for rapid achievement of desired steady-state serum concentrations, with follow-up dosing prescribed to maintain adequate therapeutic levels. Teicoplanin is an attractive outpatient parenteral antimicrobial therapy (OPAT) option; it provides activity comparable to vancomycin, with a lower reported incidence of adverse effects, a wider therapeutic window and less therapeutic monitoring.^1^ Teicoplanin provides Gram-positive coverage, targeting staphylococci (including methicillin-resistant strains) as well as streptococcal and enterococcal pathogens. Staphylococci and streptococci are the most common causative organisms of osteomyelitis, joint infections, skin and soft tissue infections and endocarditis; conditions which often can necessitate prolonged IV therapy of up to 6 weeks, representing significant healthcare costs.^2^

Teicoplanin trough serum concentration is a key predictor of clinical outcome in OPAT. The optimal dosing range for teicoplanin is extrapolated from pathogen MIC value; the optimum AUC/MIC target is debated but values >345 to >900 have been demonstrated to increase bactericidal activity of teicoplanin in haematology patients and invasive *Staphylococcus aureus* infections respectively.^3–5^ There was a strong correlation between the teicoplanin AUC and trough concentration after loading, thus trough level can be used as a proxy; trough levels of 10 mg/L and 20 mg/L correlate with AUCs of 421 mg·h/L and 707 mg·h/L, respectively.^6^ Teicoplanin breakpoints of 2 mg/L for *S. aureus* and *Enterococcus* spp. and 4 mg/L for CoNS necessitate high trough levels to obtain the desired target AUC/MIC. For pathogens with lower MIC (<2 mg/L), teicoplanin trough levels of 10–20 mg/L may be adequate. Routine MIC values are not available for all pathogens thus the high trough targets of >20 mg/L are advised in local teicoplanin dosing guidelines and in line with national recommendations.^7^

A once daily teicoplanin (ODT) regimen has demonstrated effectiveness in several studies in terms of maintaining adequate therapeutic trough concentrations. This protocol has been associated with a cure/improvement rate surpassing 90%.^8^^,^^9^ Due to teicoplanin’s long half-life (between 47 and 182 h in patients with normal renal function), it has been hypothesized that teicoplanin could be administered thrice weekly without impacting efficacy.^10^ This would further optimize the ambulatory treatment of specific conditions and yield significant benefits for both the patient and the healthcare provider compared with once daily regimens. For the patient, reduced disruption to daily life would be anticipated. For healthcare systems, fewer administration appointments would be advantageous given limited hospital resources and would maximize overall treatment capacity.^11–13^

Thrice weekly teicoplanin (TWT) dosing has previously been evaluated.^9^^,^^14–19^ Largely, it has been found to be non-inferior to ODT in outcomes and propensity to achieve target concentrations. No significant differences in incidence of adverse events (AEs) have been reported, although these studies have used differing targets for trough concentration. Lamont *et al.*^15^ reported a 93% success rate with thrice weekly administration, and an analysis of TWT for MRSA osteomyelitis cited a cure rate of 90%, with all subjects showing improvement.^9^ Regarding trough concentration, trough levels equivalent to those targeted in ODT can be attained with TWT, using clinically feasible doses.^20^ However, this seemingly viable regimen has not been widely adopted by healthcare providers, perhaps due to persisting limitations in the evidence base including the use of dosing outside of the product licensing and outside of national formulary recommendations.

Previous investigations into TWT have employed empirical dosing, later adjusted as necessary to assure attainment of target trough concentrations.^14–19^ Alternatively, using parameters that may influence trough concentration, e.g. renal function and albumin level, to tailor maintenance dosing patient-by-patient has been postulated. If effective, this would optimize the time taken to reach a therapeutic trough concentration and mean that fewer dose adjustments would be required. Lamont *et al*.^15^ conducted population pharmacokinetic analysis to generate thrice weekly dosing guidelines, and in predictive models implied that targeting therapy by renal function and body weight leads to an improvement in the proportion of target trough concentrations.

We undertook a retrospective observational study in a multicentre teaching hospital to compare a novel thrice weekly teicoplanin dosing regimen with licensed once daily regimen for treatment of adult patients on an OPAT service.

## Methods

### Study setting and design

An observational, retrospective, single-centre study was performed across multiple hospitals of the Chelsea and Westminster NHS Foundation Trust (London, UK) between March 2018 and July 2020. All adults (>18 years) receiving parenteral teicoplanin for greater than 5 days were included. Patients without therapeutic drug monitoring were excluded. Where patients received multiple teicoplanin treatment episodes, the first episode only was included. Electronic patient records (Millenium^®^, Cerner Corp., USA and ICNet^®^, Baxter, UK) and microbiology laboratory data (Sunquest ^®^ v8.3) were interrogated to identify demographic details, clinical data and outcomes. Patient demographics, pathology (urea and electrolytes including renal function, liver function tests, full blood counts), microbiology data and treatment outcomes were extracted.

### Laboratory technique

Serum teicoplanin analysis was completed at the National Antimicrobial Reference Laboratory (Bristol, UK). Trough levels were collected after a minimum 7 days of effective treatment. Microscopy for causative pathogens was investigated in line with the national UK Standards for Microbiology Investigations from PHE^21^ using the relevant media, atmospheres and duration noted in the relevant standard operating procedure. Isolate speciation was performed using MALDI-TOF MS (Biotyper^®^, Bruker). Antimicrobial susceptibilities were determined by disc diffusion using EUCAST (v. 10.0) criteria.^22^

Baseline and in-treatment blood chemistry results were analysed. Serum creatinine and estimated glomerular filtration rate (eGFR) were monitored to assess initial dosing appropriateness. Baseline albumin was recorded to assess the impact of protein binding on trough levels.

### Definitions

Teicoplanin loading occurred as 6–12 mg/kg every 12 h for 3–5 doses or a total daily dose of 18 mg/kg for 3 days. A total loading dose was calculated as the total teicoplanin dose (mg/kg) administered during the 72 h period prior to commencement of maintenance teicoplanin therapy. Maintenance teicoplanin dosing, recommended as 6–12 mg/kg daily or 16–20 mg/kg thrice weekly with dose adjustments for renal dysfunction, was measured for all included patients. The target teicoplanin concentration (trough) was defined as 20–50 mg/L.

Treatment outcomes were defined by the OPAT multidisciplinary team using an abbreviated BSAC National Outcomes Registry (NORS) score^23^ at the end of each individual treatment course. Outcome was defined as ‘success’ if patients completed OPAT treatment with or without stepdown to oral antibiotics, with complete resolution of infection and no evidence of readmission or relapse. ‘Failure’ was defined as the progression or non-response of infection despite OPAT, requiring admission or surgical intervention, or death for any reason before completion.

### Statistical analysis

Median and IQR were used to describe continuous data. Univariate analysis on non-parametric data was performed using Kruskal–Wallis/Mann–Whitney *U* test to evaluate continuous variables between groups and Wilcoxon test for comparing paired tests. Fisher’s exact test was used to evaluate categorical data. *P* values <0.05 were considered statistically significant. Data were recorded in Microsoft Excel^®^. GraphPad Prism^®^ 8.1.1 software was used for univariate analysis and to generate graphical data.

### Study approval

All data were anonymized and analysed in Excel 2017. Ethical consent was waived for this retrospective analysis following review by the Trust’s clinical governance team and this was registered as a service evaluation project. Informed patient consent was not required for this study. All data collected are stored in concordance with the Data Protection Act and the General Data Protection Regulation (GDPR) and anonymized as soon as practical to do so.

### Availability of data and materials

The datasets analysed during the current study and further details on gaining access to the intervention reported within this study are available from S.H. (stephen.hughes2@chelwest.nhs.uk) on reasonable request, as long as this meets local ethics and research governance criteria.

### Consent for publication

No data necessitating consent were used in this study.

## Results

A total of 82 patients treated with teicoplanin-based therapy in the OPAT service from March 2018 to July 2020 were included; 53/82 received thrice weekly dosing and 29/82 once daily dosing. No differences in baseline age, sex, total body weight, eGFR and serum albumin were evident between the two groups (Table 1). The once daily dosed teicoplanin group were more likely to receive combination antimicrobial therapy (20/29 versus 13/53; *P *=* *0.0001) and treat non-bone and joint infections (15/29 versus 12/53, *P *=* *0.0131). The median duration of therapy of 40 and 37 days in the once daily and thrice weekly dosed teicoplanin groups, respectively, was similar.

**Table 1.** Baseline characteristics and treatment of patients receiving once daily and thrice weekly teicoplanin OPAT therapy

	Once daily dosing	Thrice weekly dosing	Test of difference^a^
No. of patients	29	53	
male, *n* (%)	20 (70)	27 (50.9)	*P *=* *0.1614
Age, years, median (IQR)	60 (54–72)	61 (52–71)	*P *=* *0.9905
Body weight, kg, median (IQR)	80 (70.25–100)	78 (62.6–85)	*P *=* *0.1176
eGFR, mL/min, median (IQR)	89 (70–90)	85 (71–90)	*P *=* *0.7583
Serum albumin, g/dL, median (IQR)	34 (28–36)	33 (28–39)	*P *=* *0.6751
Infection source, *n*			
bone and joint	14	41	*P *=* *0.0131
skin and soft tissue	7	8	n/a
line-related infection	0	2	n/a
cardiac infection	2	1	n/a
urinary infection	2	0	n/a
intra-abdominal infection	4	1	n/a
Causative pathogen, *n*			
* S. aureus*	8	19	*P *=* *0.4742
CoNS	5	12	n/a
* Enterococcus* spp.	4	5	n/a
* Streptococcus* spp.	1	3	n/a
empirical	10	13	n/a
other	1 (*Corynebacterium* sp.)	1 (*Propionibacterium acnes*)	n/a
Treatment, *n/N*			
teicoplanin monotherapy	9/29	40/53	*P *=* *0.0001

n/a, not applicable.

a Univariate analysis on non-parametric data was performed using Kruskal–Wallis/Mann–Whitney *U* test to evaluate continuous variables between groups and Wilcoxon test for comparing paired tests. Fisher’s exact test was used to evaluate categorical data. *P* values <0.05 were considered statistically significant.

Dosing was in line with local teicoplanin dosing guidelines adjusted for patient’s body weight and renal function at initiation. The mean accumulative loading dose administered within the first 72 h was similar in the two groups (48 and 50 mg/kg respectively), administered as 12 mg/kg every 12 h for 3–5 doses (total 48–60 mg/kg) or 18 mg/kg every 24 h for 3 doses (total 54 mg/kg) depending on ambulation status at time of loading. The once daily loading was used predominantly in patients ambulating during loading.

Median teicoplanin trough level was similar in both groups (26.2 mg/L and 25.8 mg/L in once daily and thrice weekly groups, respectively [*P *=* *0.8895]) (Table 2). Patients with eGFR >90 mL/min had significantly lower teicoplanin levels in the thrice weekly group (mean [±SD] 21.4 mg/L [±10.1] versus 29.7 mg/L [±14], *P *=* *0.0178) but a non-significant trend in the once daily dosed group (mean [±SD] 28.2 mg/L [±9.4] versus 23.7 mg/L [±9.9], *P *=* *0.2201). A higher proportion of patients obtained the desired therapeutic range (20–50 mg/L) at first sampling in the once daily group (22/29 versus 26/53, *P *=* *0.0074). Supratherapeutic levels of 50.8, 53.3, 60.2 and 62.6 mg/L were obtained in four patients on thrice weekly dosing; none was associated with toxicity and dose adjustments were made on two of these patients. Subtherapeutic dosing (<10 mg/L) was identified in two patients on once daily dosing (8.6 mg/L and 9.6 mg/L); no association with therapeutic levels and clinical outcome were identified in either treatment group. No significant difference in NORS-defined treatment outcomes were identified between the two groups, with 25/29 (86.2%) and 50/53 (94.3%) success rates measured in the once daily and thrice weekly groups, respectively (*P *=* *0.2370).

**Table 2.** Teicoplanin dosing and trough concentrations in the once daily and thrice weekly dosing groups

	Once daily dosing, *N *=* *29	Thrice weekly dosing, *N *=* *53	Test of difference^a^
Loading dose, mg/kg, median (IQR)	48 (44–56)	50 (45–57.1)	*P *=* *0.3075
Maintenance dose, mg/kg, median (IQR)	12 (10–12)	16 (14.0–17.9)	n/a
Duration of therapy, days, median (IQR)	40 (17–42)	37 (24–37)	*P *=* *0.7878
Trough concentration, mg/L, mean (±SD)	26.2 (±9.6)	25.8 (±12.9)	*P *=* *0.8895
Trough concentration, *n*			
<10 mg/L	2	0	n/a
10–20 mg/L	5	23	n/a
20–50 mg/L	22	26	n/a
>50 mg/L	0	4	n/a
Trough concentration, mg/L, mean (±SD), *n*			
serum albumin			
<25 g/dL	24.9 (±10.2), *n *=* *6	33.5 (±14.9), *n *=* *8	n/a
≥25 g/Dl	26.5 (±9.8), *n *=* *23	24.4 (±12.2), *n *=* *45	n/a
renal function (eGFR)			
<90 mL/min	28.2 (±9.4), *n *=* *16	21.4 (±10.1), *n *=* *25	n/a
≥90 mL/min	23.7 (±9.9), *n *=* *13	29.7 (±14), *n *=* *28	n/a
total body weight			
<80 kg	22 (±9.5), *n *=* *14	24.4 (±12.2), *n *=* *28	n/a
≥80 kg	30.1 (±8.5), *n *=* *15	27.4 (±13.6), *n *=* *25	n/a
Treatment outcomes, *n/N* (%)			
NORS-defined improvement/cure	25/29 (86.2)	50/53 (94.3)	*P *=* *0.2370

*n* indicates number of patients.

n/a, not applicable.

a Univariate analysis on non-parametric data was performed using Kruskal–Wallis/Mann–Whitney *U* test to evaluate continuous variables between groups and Wilcoxon test for comparing paired tests. Fisher’s exact test was used to evaluate categorical data. *P* values <0.05 were considered statistically significant.

In the thrice weekly group, patients with a measured trough level taken following a 2 day dosing gap had a non-significant lower trough level (22.5 mg/L [±8.2] versus 27.4 mg/L [±14.4], *P *=* *0.2055) than those with a single day dosing gap. Local practice recommends analysing trough levels following a two day dosing gap to assess the lowest available serum level; despite this recommendation, most patients had serum level analysed after a single day dosing gap (36/53).

In patients weighing <80 kg, the once daily dosing regimen was associated with a statistically lower trough level (21.9 mg/L [±9.46] versus 30.1 mg/L [±8.5], *P *=* *0.0221) but this was not evident in the thrice weekly group (24.35 mg/L [±12.3] versus 27.42 mg/L [±13.6], *P *=* *0.3914).

Hypoalbuminaemia (<25 g/dL) was not associated with predicted reduced teicoplanin trough level in the thrice weekly (33.5 mg/L [±14.9] versus 24.4 mg/L [±12.2], *P *=* *0.0649) or once daily groups (24.9 mg/L [±10.2] versus 26.5 mg/L [±9.8], *P *=* *0.7294).

## Discussion

This retrospective observational analysis demonstrates similar therapeutic outcomes with thrice weekly and once daily administered teicoplanin in the OPAT setting. Following initiating loading, the use of thrice weekly teicoplanin enables convenient administration whilst providing adequate serum teicoplanin concentrations in the majority of patients. A high rate of NORS-defined clinical cure or improvement was reported across the study irrespective of administration frequency or therapeutic levels.

Clinical reported outcome was not inextricably linked with the chosen target range. Teicoplanin levels below 20 mg/L, defined within this study as subtherapeutic, were common in the once daily and thrice weekly administration groups (25.9% and 43.4%, respectively). The study defined therapeutic range (20–50 mg/L) is derived from the national antimicrobial reference laboratory yet lower reference ranges have been used in similar clinical studies. Lamont *et al.*’s^15^ work on thrice weekly teicoplanin targeted a trough level of 10–20 mg/L for non-severe infection and 20–30 mg/L for deep-seated or severe infections, with supratherapeutic levels defined as exceeding 60 mg/L. If these therapeutic definitions are used, all patients within the thrice weekly dosing group achieved satisfactory therapeutic levels. Lower trough levels (10–20 mg/L) may be clinically appropriate for pathogens with lower MIC values (<2 mg/L). Two patients within the once daily dosing group had trough levels below the minimum trough level of 10 mg/L.

This study used a novel teicoplanin dosing algorithm which adjusts thrice weekly maintenance dosing based on both weight and renal function. This allows for greater personalization of dosing adjusted to key pharmacokinetic properties. No maximum or capped dosing in extremes of body weight is recommended; a patient weighing 140 kg received 2 g IV thrice weekly with trough level of 11.3 g/L. Dosing in high body weights (>80 kg) was associated with equivalent trough levels to low weight patients using the thrice weekly dosing guidance. Low patient numbers with extremes of body weights were included in each arm, insufficient to make robust conclusions on optimal dosing in obesity. We recommend initial dose adjustments based on actual body weight and renal function with follow-up therapeutic drug monitoring to correct for any sub- or supratherapeutic levels.

Renal clearance accounts for the majority of teicoplanin excretion from the body. Dose adjustments of teicoplanin in patients with renal dysfunction are well established in practice and local dosing guidance adjust for chronic dysfunction. Augmented renal function, a creatinine clearance ≥120 mL/min resulting in hyperfiltration and increased teicoplanin clearance, is less well understood. In patients on daily dosed teicoplanin, the augmented renal function is less critical for therapeutic dosing. In thrice weekly administration, the extended interval between dosing can result in significantly lower trough levels in this subgroup of patients. In our study, eGFRs exceeding 90 mL/min were associated with lower trough levels. Careful therapeutic drug monitoring is advised and a dose increase (20–25 mg/kg thrice weekly) or reverting to once daily dosing should be considered in patients with subtherapeutic dosing associated with augmented renal clearance or creatinine clearance ≥120 mL/min.

The optimum time to take the teicoplanin trough level for thrice weekly administration is unclear. The longest interval between dosing (2 day dosing gap between Friday and Monday dosing) is advised locally to determine the lowest observed serum level. In practice, patients with a trough level taken after a 2 day interval had a non-significant lower trough level than those with a standard 1 day dosing gap (22.5 mg/L versus 27.4 mg/L). The long half-life of teicoplanin in the maintenance phase likely accounts for these similar trough levels despite prolonged dosing interval. Whilst outside the scope of this study, exploration of twice weekly administration at adjusted dosing may be feasible in some patients particularly with renal dysfunction.

Our study has several limitations. The retrospective design of our study inevitably reduces control over multiple confounders and data collection. The two groups were imbalanced in the nature of infection, with more orthopaedic-related infections in the thrice weekly dosed group and more polymicrobial infections requiring multiple IV therapies seen with the once daily group. This is an expected limitation due to the small sample size and heterogeneous nature of presenting infections to the OPAT service. To overcome this limitation, the primary reported outcome for this study was the therapeutic drug levels. Whilst the nature of infection may impact upon clinical cure, the pharmacokinetic parameter measured is expected to be independent to concurrent antibacterial and pathogens present.

Clinical cure definitions, in line with NORS, are recorded on completion of treatment. No routine follow-up of patient care is completed, and it is possible delayed treatment failure may have occurred with teicoplanin therapy. This limitation exists with all of our patients treated with OPAT and we acknowledge cure rates may be over-reported due to lack of long-term patient follow-up. In this comparator study, both groups are compared equally so inter-group variation is not expected to occur.

MIC values were not reported for pathogen-targeted therapy therefore optimum AUC/MIC calculations could not be assessed. Patients with low MIC pathogens may benefit with lower trough levels (10–20 mg/L) and still achieve the desired clinical outcome. Additionally, the correlation with AUC and trough level has been demonstrated with patients established on once daily dosed teicoplanin. Fewer data are available to correlate thrice weekly administered teicoplanin trough levels with the respective AUC.

Inconsistent timing of trough levels taken in respect to the 1 versus 2 day dosing gap in thrice weekly teicoplanin may unintentionally skew results. The trough level is advised to be measured following the longest dosing gap each week yet in practice this advice is poorly adhered to. The long half-life of teicoplanin is not expected to result in a significant drop in teicoplanin levels, however the study was not able to confirm this. Future studies are required with biweekly teicoplanin trough levels to assess the differences, if any, in trough levels after a 1 and 2 day teicoplanin dosing gap.

### Conclusions

In this retrospective observational study, the use of thrice weekly administered teicoplanin was associated with acceptable therapeutic trough levels and similar clinical outcomes to patients treated with a once daily administration. The use of less frequent administrations reduced the number of patient interactions (from seven to three per week), increasing capacity for healthcare professionals and reducing patient disruption on OPAT. Dosing teicoplanin thrice weekly, adjusted for renal function and total body weight, provides an effective alternative for patients requiring prolonged teicoplanin therapy. Therapeutic drug monitoring is advised for all patients on extended teicoplanin therapy due to the considerable inter-patient variability expected.

## Transparency declarations

L.S.P.M. has consulted for bioMérieux (2013–21), DNAelectronics (2015–18) and Dairy Crest (2017–18); received speaker fees from Profile Pharma (2018) and Pfizer (2018–21); received research grants from the National Institute for Health Research (2013–19), Leo Pharma (2016) and CW+ Charity (2018–21); and received educational support and speaker fees from Eumedica (2016–21). N.M. has consulted for Pfizer (2019–21) and received educational support from Eumedica (2015) and Baxter (2017). S.H. has received educational support from Baxter (2017), consulted for Shionogi (2020) and received speaker fees from Pfizer (2020-21). All other authors: none to declare.
